# A Narrative Review of the Association between Post-Traumatic Stress Disorder and Obstructive Sleep Apnea

**DOI:** 10.3390/jcm11020415

**Published:** 2022-01-14

**Authors:** Catherine A. McCall, Nathaniel F. Watson

**Affiliations:** 1 Department of Pulmonary, Critical Care and Sleep Medicine, VA Puget Sound Health Care System, Seattle, WA 98108, USA; 2 Department of Psychiatry and Behavioral Sciences, University of Washington School of Medicine, Seattle, WA 98195, USA; 3 Department of Neurology, University of Washington School of Medicine, Seattle, WA 98195, USA; nwatson@uw.edu; 4 University of Washington Medicine Sleep Center, Seattle, WA 98104, USA

**Keywords:** post-traumatic stress disorder, obstructive sleep apnea, PTSD, OSA

## Abstract

Obstructive sleep apnea (OSA) and post-traumatic stress disorder (PTSD) are often co-morbid with implications for disease severity and treatment outcomes. OSA prevalence is higher in PTSD sufferers than in the general population, with a likely bidirectional effect of the two illnesses. There is substantial evidence to support the role that disturbed sleep may play in the pathophysiology of PTSD. Sleep disturbance associated with OSA may interfere with normal rapid eye movement (REM) functioning and thus worsen nightmares and sleep-related movements. Conversely, hyperarousal and hypervigilance symptoms of PTSD may lower the arousal threshold and thus increase the frequency of sleep fragmentation related to obstructive events. Treating OSA not only improves OSA symptoms, but also nightmares and daytime symptoms of PTSD. Evidence suggests that positive airway pressure (PAP) therapy reduces PTSD symptoms in a dose-dependent fashion, but also presents challenges to tolerance in the PTSD population. Alternative OSA treatments may be better tolerated and effective for improving both OSA and PTSD. Further research avenues will be introduced as we seek a better understanding of this complex relationship.

## 1. Introduction

Post-traumatic stress disorder (PTSD) is a psychiatric illness occurring after exposure to a traumatic event, in which the individual experiences intrusive memories such as flashbacks, avoidance of trauma reminders, negative changes in mood and cognition, hyperarousal symptoms, and impairment of social, occupational, and interpersonal functioning [1]. Sleep-related symptoms may be particularly prominent and feature nightmares about the event, fear of sleep, night terrors, insomnia, and dream-enactment behavior which often involves thrashing and fighting [2]. Although lifetime trauma exposure estimates worldwide are as high as 70%, PTSD develops in a relatively smaller segment of the population with 8.3% lifetime prevalence [3]. PTSD prevalence also varies considerably with other factors such as the number of trauma exposures and the severity of the trauma [4]. PTSD may resolve in some individuals within a period of weeks to months, whereas in others it becomes a chronic condition [5,6]. One study of rape survivors assessed for PTSD diagnosis every week after their assault found that 94% met criteria for PTSD at the first assessment, 65% met criteria about one month later, and 47% met criteria about three months later. The authors noted a distinct difference between subjects whose PTSD improved over time and those who remained symptomatic [5]. Much attention has been placed in recent years on identifying modifiable factors that may contribute to developing a persistent post-traumatic syndrome resilience protecting against this disorder.

Research over the past decade has uncovered significant evidence for the bidirectionality of sleep problems and PTSD. As many as 70% of PTSD sufferers experience some kind of sleep disturbance [2]. About 41% of individuals with PTSD report trouble initiating sleep versus 13% of those without PTSD, and 47% of PTSD sufferers report difficulty maintaining sleep [7]. Sleep disturbances may also include nightmares as well as dream enactment behaviors that have led to a proposed new sleep disorder, trauma-associated sleep disorder [8,9,10]. Sleep-related problems were traditionally considered to be symptoms of the disorder rather than separate diagnoses. However, many studies have now suggested that sleep problems preceding a traumatic experience may predict or even predispose to the development of persistent stress and PTSD [11,12,13,14].

Compared with patients without sleep complaints, PTSD patients with comorbid sleep disorders experience higher rates of substance abuse, depression, and suicidality [15,16,17]. Sleep disturbances are predictors of PTSD treatment nonresponse, and may continue even after successful treatment of daytime PTSD symptoms [18,19,20,21,22,23] This underscores the importance of identifying and managing sleep problems in this population. Treating sleep problems such as nightmares and insomnia is also now understood to improve daytime symptoms of PTSD [24,25] While this may not be surprising given that nightmares and sleep difficulties are core symptoms of PTSD diagnostic criteria, what has been surprising is the more recent evidence showing similar bidirectionality in PTSD and obstructive sleep apnea (OSA) diagnoses and treatment [26,27,28]. The following sections will highlight research in these domains.

## 2. The OSA and PTSD Overlap

OSA is a disorder of recurrent partial or complete upper airway collapse during sleep, leading to snoring, reduced ventilation, intermittent and cumulative hypoxemia, frequent arousals from sleep, and alterations in sleep architecture. OSA diagnosis requires a sleep study showing at least five predominantly obstructive events per hour with related symptoms, or 15 events/hour without symptoms [29]. Untreated OSA is associated with a myriad of medical and psychiatric health conditions, including cardiovascular disease [30], metabolic disorders [31], psychiatric disorders [32], motor vehicle accidents [33] and all-cause mortality [34]. The prevalence of OSA is approximately 13% in men and 6% in women aged 30–70 years when using an apnea-hypopnea index (AHI) cutoff of 15 events per hour, whereas 14% of men and 5% of women meet the criteria of AHI > 5 with symptoms of daytime sleepiness [35]. The risk of OSA is higher in individuals with elevated body mass index (BMI), male sex, age > 50 years, neck circumference > 40 cm, and in post-menopausal women [36,37].

A significant co-prevalence has been found between OSA and PTSD. This association has been confirmed in over a dozen studies to date encompassing both military and civilian populations, with a recent meta-analysis of 12 studies showing pooled prevalence rates of 75.7% for AHI ≥ 5 and 43.6% for AHI ≥ 10 [38], Individuals with OSA and PTSD (OSA + PTSD) may demonstrate fewer “typical” features of OSA such as elevated BMI and older age [16,39]. One study found that age- and sex-matched patients with sleep-disordered breathing and trauma exposure had lower BMI, less snoring, and more insomnia, nightmares, psychotropic medication use, leg jerks, and upper airway resistance findings in PSG than sleep-disordered breathing patients without trauma exposure [39]. Patients with OSA + PTSD have worse symptoms of both disorders than those suffering from either disorder alone, reporting lower quality of life and more somnolence compared to those with OSA only [40], and worse nightmares, sleep quality, anxiety, depression, posttraumatic stress, and quality of life compared to those with PTSD only [16].

There are multiple hypotheses regarding why individuals with PTSD experience significantly higher rates of OSA than the general population. One possibility is that the arousal threshold is lowered in PTSD due to a hyperactive chronic stress response, leading to greater arousal sensitivity to mild obstructive events and thereby increased frequency of hypopneas and respiratory effort-related arousals (RERAs). There is additionally evidence that sleep fragmentation and sleep deprivation may promote airway collapse, which may then further increase arousals [41]. PTSD-triggered sleep fragmentation resulting from hyperarousal processes may also result in lighter sleep, which is associated with respiratory instability [27,42,43,44]. A low arousal threshold has been found to have high prevalence in veterans with OSA [45] and PTSD [46].

Conversely, upper airway collapse occurring during sleep may have multiple effects that predispose to persistent stress symptomatology. OSA is often worse during rapid eye movement (REM) sleep due normal atonia of accessory respiratory muscles that would otherwise aid in opening the upper airway during collapse. Obstructive events occurring during REM sleep may precipitate increased frequency of arousals that may heighten nightmare awareness and recall. Additionally, awakenings from obstructive events often occur with sympathetic nervous system activation, with associated symptoms of racing heart, shortness of breath, and anxiety, all of which could worsen the experience of a nightmare [47]. OSA has been associated with nightmares even in individuals without PTSD, often with content related to suffocation, choking, drowning, strangulation, burial, and death [48,49,50,51]. A study of patients with OSA who slept in a laboratory before and after starting continuous positive airway pressure (CPAP) were awakened after the beginning of every REM period for dream reports. Dream recall was increased after obstructive events, with these dreams being significantly more negative than dreams occurring without obstructive events [52]. Carrasco et al. hypothesized that respiratory events occurring during REM sleep lead to stimulation of the limbic system, which may increase the emotional content of dreams [53].

In the setting of a traumatic stress, OSA-induced REM sleep fragmentation may also interfere with functions of REM sleep itself that are critical for healthy processing of emotional memories. Substantial behavioral and neurophysiological evidence supports the understanding that sleep contributes to emotional memory consolidation, and sleep deprivation negatively impacts it [54,55,56,57]. REM sleep in particular appears to play an important role in the acquisition and long-term retention of appropriate fear learning, including the ability to discriminate between threatening and non-threatening stimuli [54,55,58]. REM sleep deficiency is associated with next-day emotional reactivity and amygdala responsivity [54,59,60]. REM sleep is also associated with fear extinction memory performance, as well as fear inhibition [61,62]. Accordingly, disturbed REM sleep is associated with impairments in both conditioned fear and extinction learning [63,64,65]. Similar impairments in fear learning and emotional dysregulation have also been found with PTSD [66,67], along with evidence of REM sleep abnormalities [59,68,69]. This body of evidence supports hypotheses that REM sleep disturbance may play a role in the development and persistence of PTSD.

The association between PTSD and OSA is additionally supported by studies finding that OSA diagnosis prior to trauma exposure also predicts the development of PTSD [2,28]. Furthermore, individuals with comorbid PTSD and OSA show impairments in fear discrimination, inhibition, and extinction that improve with OSA treatment [70]. OSA occurring during REM sleep could thus be implicated as a potential cause of REM sleep fragmentation and contribute to PTSD pathology. These data in total strongly suggest that there is likely a bidirectional relationship between OSA and PTSD in which each condition mutually reinforces the other [26,27,28,59]. The cycle of stress-induced arousals and insomnia promotes sleep fragmentation, which worsens PTSD symptoms.

The implications for the role of OSA in PTSD may reach beyond the development of post-traumatic stress symptoms. The most effective treatments for PTSD are recognized to be trauma-focused psychotherapies, including exposure-based therapies such as prolonged exposure (PE) therapy and cognitive processing therapy (CPT) [22,71,72,73]. These therapies rely on the patient’s ability to effectively generalize extinction memory. Sleep-disordered breathing has been found to negatively impact the effectiveness of PE, possibly by disrupting the extinction memory consolidation and generalization that normally occurs during sleep [23]. OSA may thus not only play a role in the development and persistence of PTSD, but also hamper the effectiveness of core therapies to treat it.

Research on treatment strategies for addressing sleep concerns has yielded insight into alternative treatments to improve PTSD severity and outcomes. Successful treatment of nightmares and insomnia is known to have a positive impact on daytime PTSD symptoms [25,74]. As our understanding of the OSA+PTSD connection has developed, a growing body of evidence has suggested that treating OSA may also improve PTSD symptoms. There are several potential advantages to targeting PTSD symptoms using sleep treatments. Despite the evidence base demonstrating effectiveness of PTSD psychotherapies, 13–39% of patients prematurely discontinue exposure therapy for PTSD [71] and 20% of veterans do not experience clinically significant improvement after PE [72]. Possible reasons for this include difficulty tolerating trauma-related memories and emotions, and/or stigma of receiving mental health treatment. Studies in veteran populations have shown that individuals with PTSD report greater willingness to seek sleep medicine treatments over explicitly PTSD-focused care [75,76]. The following sections detail the benefits and challenges of addressing PTSD-related symptoms through treatment of OSA.

## 3. Impacts of OSA Treatment on PTSD

### 3.1. PAP Therapy

The cornerstone of OSA treatment is preventing collapse of the upper airway during sleep. As such, the current gold-standard treatment of OSA is PAP therapy [77,78]. CPAP devices deliver pressurized room air through a hose and mask in order to splint the upper airway. This effectively prevents obstruction by the tongue, soft palate and surrounding tissues, enables normal respiratory processes, and minimizes respiratory-related arousals. CPAP has demonstrated clinically significant reduction in disease severity, daytime sleepiness, hypertension, motor vehicle accidents, and quality of life [78]. Meta-analyses of treatment impacts on cardiovascular events, cognitive function and mood have produced mixed results [78,79,80].

Studies on the effects of CPAP in patients with OSA+PTSD have shown improvement in PTSD-related symptoms in those who were adherent to CPAP. (Table 1) In 1998, a case report was published in which a Veteran with OSA+PTSD experienced dramatic improvement in sleep quality, nightmare frequency, and daytime sleepiness after starting CPAP therapy [81]. A retrospective review in 15 patients in 2000 found that 75% of those using CPAP reported improvement in PTSD symptoms, while those who declined CPAP experienced worsening symptoms [82]. A larger study in 2014 not only found that CPAP improved nightmares, but also noted a dose-dependent relationship, with every 10% improvement in CPAP adherence decreasing the mean number of nightmares by one nightmare/week [83]. El-Solh et al. likewise found that an increased number of hours/night using CPAP was associated with improvement in PTSD symptoms in veterans. Subjects with severe OSA experienced greater improvements in PTSD symptoms than those with mild to moderate OSA over a period of 3 months, but both groups reported reduced nightmare distress and nightmare frequency [84]. Veterans with PTSD and a new diagnosis of OSA reported significant reductions of PTSD symptoms, sleepiness, and depressions, as well as improved sleep quality, daytime functioning, and quality of life over a period of 6 months [85]. A study of Veterans with PTSD and subclinical PTSD found that CPAP therapy reduced PTSD symptoms and nightmare frequency in both groups, though the subclinical PTSD group required higher adherence to achieve symptom improvement. Poor adherence to CPAP resulted in increased PTSD Checklist (PCL) scores in the subclinical PTSD group, and the authors speculated that untreated OSA may predispose to the development of overt PTSD [86].

Despite the well-documented improvements in PTSD-related symptoms with OSA treatment, several notable challenges occur in this population. Adherence to PAP therapy is problematic in the general population, with 29–83% of patients using CPAP under 4 h per night [87]. Adherence is significantly lower in the PTSD population. The meta-analysis of Zhang et al. found three studies showing that patients with PTSD demonstrated significantly lower adherence to PAP therapy, including regular use and average duration of use per night, compared to those with OSA alone [38]. One study showed 40% adherence in PTSD patients after 30-day follow-up, versus 70% adherence in non-PTSD controls. Reasons given for non-adherence included factors that were similar between PTSD and non-PTSD groups, including mask discomfort, air hunger, and high pressure; claustrophobia was reported slightly more frequently in the PTSD group. Nightmares and absence of sleepiness were also predictors of non-adherence [88].

**Figure 1:**
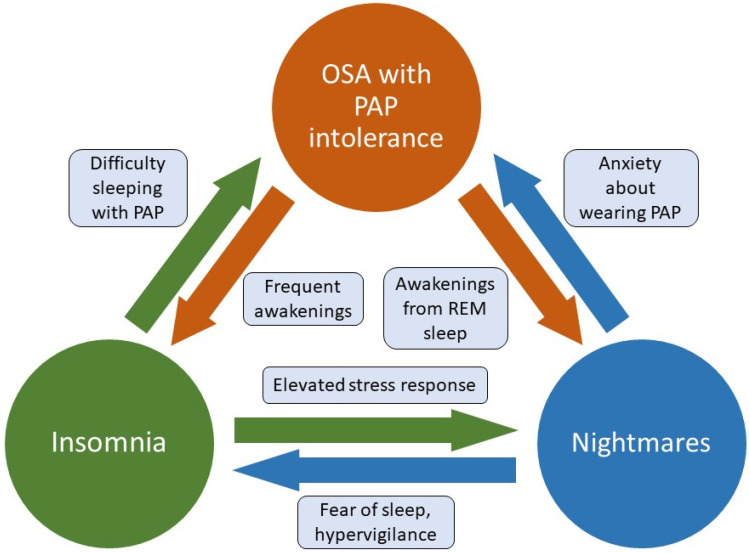
Factors contributing to untreated OSA, insomnia, and nightmares in PTSD sufferers. Untreated sleep apnea may lead to frequent awakenings which precipitate and/or perpetuate insomnia, as well as arousals from REM sleep leading to increased nightmare intensity and recall. Difficulty initiating and maintaining sleep interferes with the ability to tolerate PAP therapy, and hyperarousal related to insomnia may increase nightmares via elevated stress response during REM sleep. The presence of nightmares often leads to fear of sleep, hypervigilance, and poor sleep hygiene (e.g., leaving lights on) that worsen insomnia. Nightmares may also reduce PAP tolerance due to increased anxiety and hypervigilance. OSA, obstructive sleep apnea. PAP, positive airway pressure. REM, rapid eye movement.

Anecdotally, patients with OSA and PTSD have also reported reluctance to use PAP therapy due to fear of becoming less aware or unable to respond to the potential threats in the environment, which speaks to issues arising from hypervigilance-related symptoms. Other common complaints by patients with OSA + PTSD in the clinical environment have included challenges in feeling tangled or restrained by the hosing, particularly in those with disruptive sleep behaviors. In military populations, reminders of wearing masks during military training and combat are frequent reasons cited for CPAP intolerance. The presence of comorbid anxiety and insomnia have also been speculated to contribute to difficulty sleeping while wearing a mask [88,89]. The presence of insomnia with OSA is often referred to as “comorbid insomnia with OSA” (COMISA) or “complex insomnia” and has been found to reduce PAP adherence and effectiveness due to the sufferer’s inability to initiate and maintain sleep while using PAP therapy [90]. Figure 1 shows the complexity of comorbid sleep disorders seen with PTSD, and how they may interact to complicate treatment efforts.

Even when patients adhere to treatment, those with PTSD may experience less benefit than those without PTSD. One study found that resolution of sleepiness occurred in 82% of patients without PTSD who were adherent to PAP therapy, but only 62.5% of PAP-adherent patients with PTSD. Quality of life normalization with PAP therapy was also achieved by fewer patients with PTSD (56.3%) than by those without PTSD (72%) [40]. These issues, as well as problems in tolerating PAP therapy in patients with OSA+PTSD, have led to research efforts exploring alternative treatments for OSA in this population.

### 3.2. Alternative OSA Treatments

Beyond CPAP, alternative treatments for OSA include oral appliances, positional therapy, weight loss, and surgery. Few of these treatments have been studied in the OSA +PTSD population. Oral appliances, sometimes referred to as mandibular repositioning devices (MRD) or mandibular advancement devices (MAD), are a frequently explored treatment option in those patients who cannot tolerate CPAP. MRDs are customized appliances that facilitate protrusion of the mandible, thus preventing collapse of the tongue and soft tissues of the airway. MRDs are considered more beneficial in mild to moderate cases of OSA, and may be less effective with severe OSA [91]. Studies on long-term usage have shown subjective adherence is excellent, with retained effectiveness in treating snoring and sleepiness, but with mild reductions in efficacy over time for nocturia, mouth opening, headache, unrefreshing sleep, and tiredness [92]. Long-term adherence may be affected by adverse dental effects, device features, device wear, and/or progression in severity of OSA [93,94,95,96].

El-Solh et al. used a randomized crossover trial to evaluate the effectiveness and adherence of CPAP versus MRD in Veterans with OSA+PTSD. [89] They found that the mean residual number of obstructive events after titration with each treatment was 26.3 events per hour with MRD versus 3.9 events per hour with CPAP. Overall, 71% of CPAP-titrated patients experienced complete resolution of OSA, versus 14% of those with MRDs. However, patients using MRD exhibited longer sleep time and higher sleep efficiency during the titration than those using CPAP. Adherence was improved in the MRD condition, with 58% reporting preference for MRD and 29% preferring CPAP. Despite the differences in OSA treatment effectiveness between CPAP and MRD, the effect size improvements in PTSD symptoms were comparable for both treatments. Likewise, excessive sleepiness and sleep quality also improved with both treatments, though sleep quality was more improved with CPAP. This suggests that some components of treatment effectiveness are not captured solely by reduction in AHI. As this study evaluated only short-term treatment effects, the long-term effects and adherence in this population are not known.

Other alternative OSA treatments that have been studied in PTSD patients include hypoglossal nerve stimulation (HNS). This implanted device causes contraction of the genioglossus muscle with tongue protrusion during inspiration to prevent upper airway collapse during sleep. HNS was approved in 2014 in the US for the treatment of OSA in patients meeting specific criteria for AHI, BMI, airway collapse pattern on drug-induced sleep endoscopy, and history of PAP intolerance. A study of 46 OSA patients (26 with PTSD and 17 without PTSD) who underwent HNS implantation found complete control of OSA (defined as AHI < 5 events/hour), improvement in excessive sleepiness, and mean best adherence were similar for patients with and without PTSD [97]. PTSD patients with COMISA had significantly lower adherence than those without insomnia. The authors noted that PTSD symptom questionnaire scores did not significantly change pre/post-surgery, though 5 of 7 patients showed a slight downward trend in scores.

**Table 1:** Studies evaluating OSA treatment effects on PTSD. PAP, positive airway pressure. MRD, mandibular repositioning device. HNS, hypoglossal nerve stimulation. REM, rapid eye movement sleep. NREM, non-rapid eye movement sleep. PCL-M, PTSD checklist-military. PCL-S, PTSD checklist-specific. PCL-5, PTSD checklist for the Diagnostic and Statistical Manual of Mental Disorders, Fifth Edition.

Authors	Year	Study Type	Study Population	Age (Mean Years ± SD)	Sex (% Male)	Treatment Type	Main Findings
Youakim et al. [81]	1998	Case Report	Veteran	42	100	PAP therapy	Nightmare frequency and intensity was improved after 4 months of PAP therapy, as well as daytime PTSD symptoms.
Krakow et al. [82]	2000	Retrospective	Civilians	Treatment: 43.8 ± 14.1 No treatment: 50.8 ± 14.9	Not reported	PAP therapy	PAP users reported a median 75% improvement in PTSD symptoms; subjects without PAP therapy reported worsening symptoms.
Tamanna et al. [83]	2014	Retrospective	Veterans	58 ± 12.05	97	PAP therapy	The mean number of nightmares per week was reduced over 6 months of PAP therapy. Reduced nightmare frequency was best predicted by PAP adherence.
El-Solh et al. [84]	2017a	Prospective cohort	Veterans	52.6 ± 14.2	92.5	PAP therapy	PCL-M scores improved after 3 months of PAP therapy, in a dose-dependent manner. PAP usage was the only significant predictor of overall PTSD symptom improvement.
Orr et al. [85]	2017	Prospective cohort	Veterans	52 (range 43-65)	87.5	PAP therapy	PCL-S scores improved over 6 months of PAP therapy. The percentage of nights in which PAP was used, but not mean hours used per night, predicted improvement.
Ullah et al. [86]	2017	Prospective cohort	Veterans	51.24 ± 14.74	Not reported	PAP therapy	PCL-M scores improved after 6 months of PAP therapy in PTSD patients, whereas non-PTSD patients with low adherence showed worsening of PCL-M scores.
El-Solh et al. [89]	2017b	Randomized crossover trial	Veterans	52.7 ± 11.6	Not reported	MRD compared to PAP therapy	71% of CPAP users and 14% of MRD users had complete OSA resolution during titration studies; however MRD users had longer sleep time, higher sleep efficiency and better adherence to treatment. Both treatments showed similar improvements in PCL-M scores after 3 months.
El-Solh et al. [90]	2018	Prospective	Veterans	PTSD with comorbid OSA and insomnia: 47.2 ± 10.8	PTSD with comorbid OSA and insomnia: 72	PAP therapy	PCL-M scores improved after 3 months of PAP therapy in patients with and without insomnia. The change in PCL-M scores was smaller in those with insomnia. PAP adherence was also lower in the insomnia group.
				PTSD with OSA: 52.7 ± 9.7	PTSD with OSA: 86		
Patil et al. [97]	2021	Retrospective and prospective case series	Veterans	59.3 ± 10.6	96.2	HNS	Resolution of OSA and adherence were similar for patients with and without PTSD; adherence was lower in PTSD patients with insomnia. PCL-5 scores obtained 6–12 months after surgery did not significantly change from baseline.

Surgical options for treating OSA also include modification of the upper airway soft tissue, including the palate, tongue base, and lateral pharyngeal walls [98]. We were unable to find studies evaluating the effects of this treatment specifically in PTSD patients.

There is limited evidence for treatments that target OSA in conjunction with insomnia and/or nightmares. Some small studies have investigated the use of sedative-hypnotics to raise the arousal threshold and improve CPAP adherence, with mixed results [44,99,100,101]. This approach would necessitate caution with regards to potential risks of using sedating medications if OSA were not fully treated. Many medications, including novel agents, have not been tested as OSA monotherapy in the OSA + PTSD population [102]. Another medication consideration is that antidepressant usage (selective serotonin reuptake inhibitors or SSRIs, and serotonin-norepinephrine reuptake inhibitors or SNRIs) has been associated with higher odds of low arousal threshold, as well as risk of abnormal sleep-related movements and dream enactment behaviors [10,46,103]. This adds a further layer of complexity, given that SSRIs and SNRIs are among the first-line pharmacotherapies for the treatment of PTSD [104,105]. Managing both psychiatric and sleep problems with PTSD thus presents additional challenges. Further research is needed to determine best clinical practice for the treatment of PTSD with comorbid sleep disorders.

## 4. Conclusions

Recent decades have shown a strong, bidirectional relationship between PTSD and sleep problems, with OSA being an increasingly recognized contributor. Individuals with both OSA and PTSD experience worse symptoms of both disorders than those with only one of these illnesses. The direction of initial causality is not clear, though potential pathology may arise from sleep disturbance around the time of trauma exposure and/or stress-related hyperarousal, sleep fragmentation, and respiratory instability leading to impaired REM sleep function. Evidence-based treatments for PTSD may not address the crucial component of sleep disturbance from OSA. Research on the impact of OSA treatment in the OSA+PTSD has shown substantial clinical benefits for both disorders with PAP therapy, with the limiting factor of significant intolerance in this population. Second-line treatments, including MRD and HNS, have shown benefit with improved adherence, though studies are limited thus far. It is important to note that there is a paucity of randomized controlled trials evaluating OSA treatment effects on PTSD, particularly in women and non-veterans. Further research is needed in this area.

Many patients with PTSD suffer from multiple sleep problems, including OSA, insomnia, nightmares, and dream enactment behaviors, with each disorder presenting challenges in the treatment of the others. Future research will also optimally test combinations of treatments for these complex comorbidities.
